# Epigenetic Manipulation of Psychiatric Behavioral Disorders Induced by *Toxoplasma gondii*

**DOI:** 10.3389/fcimb.2022.803502

**Published:** 2022-02-14

**Authors:** Kun Yin, Chao Xu, Guihua Zhao, Huanhuan Xie

**Affiliations:** Shandong Institute of Parasitic Diseases, Shandong First Medical University & Shandong Academy of Medical Sciences, Jining City, China

**Keywords:** *Toxoplasma gondii*, cerebral cyst, psychiatric and behavioral disorders, epigenetic, parasite manipulation

## Abstract

*Toxoplasma gondii* is known to have a complex life cycle and infect almost all kinds of warm-blooded animals around the world. The brain of the host could be persistently infected by cerebral cysts, and a variety of psychiatric disorders such as schizophrenia and suicide have been reported to be related with latent toxoplasmosis. The infected animals showed fear reduction and a tendency to be preyed upon. However, the mechanism of this “parasites manipulation” effects have not been elucidated. Here, we reviewed the recent infection prevalence of toxoplasmosis and the evidence of mental and behavioral disorders induced by *T. gondii* and discussed the related physiological basis including dopamine dysregulation and gamma-aminobutyric acid (GABA) pathway and the controversial opinion of the necessity for cerebral cysts existence. Based on the recent advances, we speculated that the neuroendocrine programs and neurotransmitter imbalance may play a key role in this process. Simultaneously, studies in the evaluation of the expression pattern of related genes, long noncoding RNAs (lncRNAs), and mRNAs of the host provides a new point for understanding the mechanism of neurotransmitter dysfunction induced by parasite manipulation. Therefore, we summarized the animal models, *T. gondii* strains, and behavioral tests used in the related epigenetic studies and the responsible epigenetic processes; pinpointed opportunities and challenges in future research including the causality evidence of human psychiatric disorders, the statistical analysis for rodent-infected host to be more vulnerable preyed upon; and identified responsible genes and drug targets through epigenetics.

## Introduction

More than 110 years ago, *Toxoplasma gondii* was initially discovered in the tissues of a North African rodent *Ctenodactylus gundi* by Nicolle and Manceaux (1908) and was also described in the tissues of a rabbit in 1908 by Splendore in Brazil (Chen et al., 2019). *T. gondii* is one of the important parasitic protozoa belonging to Phylum Apicomplexa. It is widely spread around the world and affects almost all kinds of terrestrial and marine warm-blooded creatures in its complete life cycle. Human beings are only one of its intermediate hosts, while felids are the unique final host of *T. gondii*, and its intestinal epithelial cells are the unique location for initiating the sexual reproduction. The feces excreted by the infected cats, which contain billions of infective oocysts, become the major source of infection (Rahimi et al., 2015). Oocysts can contaminate natural water, soil, vegetables, and even hide in aerosol, and usually cause outbreaks of toxoplasmosis. For instance, an outbreak of toxoplasmosis caused by oocysts pollution in water source has been reported in Brazil (de Moura et al., 2006). For humans, individuals may be infected by consuming undercooked meat that contains cysts not thoroughly heated or by vertical transmission from mother to fetus (Jones et al., 2014b). In addition, blood transfusion, organ transplantation, and oral ingestion of dairy products are also subsistent routes to infection (Dard et al., 2018; Dubey et al., 2020; Xu et al., 2020). Therefore, *T. gondii* is a kind of parasite that is closely related to human life.

At present, the seropositive infection rate of *T. gondii* is largely fluctuating around the world. In United States, the seropositive rate of *T. gondii* is 10%–15%, but in the south center of America, Mediterranean region, Europe, and parts of Southeast Asia, the infection rate is higher than 60% (Pappas et al., 2009). In China, the seropositive rate is 0.79%–16.8% in the last two decades (Cheng et al., 2005). The peak of serum IgG antibodies to *T. gondii* could be detected in 2–3 months, and still at detectable levels after then, so it could be used to reflect a chronic infection (Robert-Gangneux and Darde, 2012). It is worth noting that the average chronic infection rate of *T. gondii* worldwide is approximately 25% (Hakimi et al., 2017), and 13.2% in the United States in individuals older than 5 years of age (Jones et al., 2014a). In China, human and animal infections have been detected at least in 30 provinces (Cheng et al., 2005). The overall prevalence of anti-*T.gondii* IgG in 2,634 healthy individuals and 547 patients with certain psychiatric disorders in Changchun, Daqing and Shanghai, China was 12.3% (Pan et al., 2017). In some other regions, the chronic infection rate is as high as 30% (Hakimi et al., 2017). Among the customary meat animals, the highest infection rate has been identified in swine, which is reaching to 39.45%, followed by sheep and chicken at 13.87% and 19.00%, respectively (Dong et al., 2018). For cats, the average infection rate has come up to 24.50% (Ding et al., 2017). The high infection rate in human beings, poultries, and domestic animals makes *T. gondii*, and *Salmonella*, *Listeria monocytogenes*, and *Escherichia coli* O157, a major food safety concern (Shurson et al., 2021).

*T. gondii* is capable of causing damages to individuals with immune deficiency such as HIV/AIDS, those who had organ transplant, and those undergoing chemotherapy (McLeod and V, 2014). However, in healthy individuals, primary infection is asymptomatic or mild, such as a mild fever, enlargement of lymph nodes, or muscle weakness. Acute infection usually could be self-healing in infected individuals with complete immunity, while tachyzoites often transform to bradyzoites and be dormant in a tissue cyst.

Tissue cysts are typically detected in the brain, muscle, and liver; can persist for the life of the intermediate host; and serve as a reservoir for recrudescence when an individual becomes immunocompromised. Significantly, tissue cysts have a predilection to infect host neural tissues, causing asymptomatic brain infection or encephalitis (Petersen, 2007). As a result, human latent toxoplasmosis is considered as a potential causative agent for a variety of psychiatric disorders especially for schizophrenia (Tyebji et al., 2019). When pregnant women are infected with *T. gondii*, the rate of vertical infection to the fetus is as high as 50%, which may lead to congenital infection and cause chorioretinitis, hydrocephalus, intracranial calcification, and even death, once the infection occurs before 24 weeks of pregnancy (Cong et al., 2015). While in the period of 26–40 gestational weeks, transmission of infection usually results in subclinical disease, but the tissue cysts carried by the fetus also serve as a reservoir for the occurrence of recrudescence and may cause chorioretinitis and encephalitis, epilepsy, and psychomotor or psychiatric retardation (Remington et al., 2001; McLeod and V, 2014). Meanwhile, *Toxoplasma* encephalitis is one of the major death triggers in AIDS patients as a consequence of recrudescence. When the immune system is suppressed, the occurrence of re-transformation of cysts into aggressive tachyzoites usually results in acute fatal toxoplasmosis (Hernandez et al., 2017). Nevertheless, the current clinical standard toxoplasmosis treatment, namely, sulfadiazine combined with pyrimethamine therapy, is able to expel parasites (Dubey, 2008) but cannot kill tissue cysts (Mccabe, 2001). Therefore, *T. gondii* infection has become an important and urgent problem both in immunodeficiency disease and psychiatric disorder.

## What Psychiatric Disorders Do Chronic *T. gondii* Infection Cause in the Host?

The study of the relationship between latent toxoplasmosis and human cognitive function could be traced to the report of Burkinshaw et al. as early as 1953 (Burkinshaw et al., 1953). In recent years, more and more evidence has shown that latent toxoplasmosis may adversely affect individuals’ cognitive function and associate with mental disorders, violence, risk taking, personality changes, and cognitive impairments. This evidence includes raised seropositivity of anti-toxoplasma antibodies among those with mental disorders, controlled behavioral analysis in humans, and experimental evidence in rodent models.

## The Well-Established Association of Psychiatric Disorders in Humans

For humans, mental and behavioral disorders are associated with multifactorial causation including genetic predisposition and environmental mediation. The neurotropic parasite *T. gondii* has aroused increasing interest because it can serve as one of the infectious agents and play an etiological role in host brain development and neuroimmune response. Consistently, there are numerous reports showing a relationship between toxoplasmosis and psychiatric disorders and personality changes; the current findings discussed in this review are summarized in **Table 1**.

**Table 1:** Selected current studies included in this review to analyze the relationship between *T. gondii* infection and human psychiatric and behavioral disorders.

Year	Author	Design	Sample size	Related psychiatric disorder type*
2015	Sutterland et al.	Meta-analysis	12,009 cases and 71,441 controls	Schizophrenia (OR = 1.81, p < 0.00001), bipolar disorder (OR = 1.52, p = 0.02), obsessive compulsive disorder (OR = 3.4, p < 0.001), addiction (OR = 1.91, p < 0.00001).
2021	Akgül et al.	Case–control	117 cases in schizophrenia and 120 cases in healthy control	Schizophrenia patients (p < 0.001) with suicide attempt (p < 0.05).
2019	Burgdorf et al.	Case–control	81,912 blood donors and 11,546 controls	Schizophrenia (OR = 1.47; 95% CI = 1.03–2.09) and traffic accident (OR = 1.11; 95% CI = 1.00–1.23).
2015	Monroe et al.	Meta-analysis	2,353 patients and 1,707 controls	Chronic psychosis (OR = 2.54, 95% CI = 1.63–3.96, p < 0.01).
2000	Flegr et al.	Case–control	230 women with toxoplasmosis	Personality changes.
2018	Gohardehi et al.	Meta-analysis	1,841 identified studies	Risk of having traffic accidents (p < 0.05).
2019	Sutterland et al.	Meta-analysis	4,229 cases and 12,234 controls in traffic accidents and 2,259 cases and 9,400 controls in suicide attempts	Risk of having traffic accidents (OR = 1.69; 95% CI = 1.20–2.38) and suicide attempt (OR = 1.39; 95% CI = 1.10–1.76).
2015	Ngoungou et al.	Meta-analysis	1,280 epilepsy 1,608 controls	Epilepsy (OR = 2.25, 95% CI = 1.27–3.9).
2019	Sadeghi et al.	Meta-analysis	3,771 epileptic patients and 4026 healthy controls	Cryptogenic epilepsy (OR = 2.65; 95% CI = 1.91–3.68) and active convulsive epilepsy (OR = 1.37; 95% CI = 1.09–1.72).
2019	Bayani et al.	Meta-analysis	301 patients and 313 controls	Alzheimer’s diseases (OR = 1.38; 95% CI = 0.99–1.92).
2021	de Haan L et al.	Meta-analysis	13,289 healthy participants	Processing speed (SMD = 0.12; 95% CI = 0.05–0.19), working memory (SMD = 0.16; 95% CI = 0.06–0.26), short-term verbal memory (SMD = 0.18; 95% CI = 0.09–0.27), executive functioning (SMD = 0.15; 95% CI = 0.01–0.28).
2020	Kamal et al.	Case–control	348 individuals and 400 controls	Depressed (OR = 2.9; 95% CI = 1.9–4.3) and actual suicide attempts (OR = 6.2; 95% CI = 3.4–11.2).
2021	Al et al.	Case–control	36 maternal, 36 from their non-autistic children, and 36 from their autistic children	Childhood autism.
2019	Flegr et al.	Internet questionnaires survey	6,367 individuals	Autism (OR = 4.78), schizophrenia (OR = 3.33), attention deficit hyperactivity disorder (OR = 2.50), obsessive–compulsive disorder (OR = 1.86), antisocial personality disorder (OR = 1.63), learning disabilities (OR = 1.59), and anxiety disorder (OR = 1.48).
2020	Soleymani et al.	Meta-analysis	15 studies included in the meta-analysis	Suicide (OR = 1.43, 95%CI = 1.15–1.78).
2020	Nayeri et al.	Meta-analysis	1,205 headache or migraine patients and 1,312 controls	Headache (OR = 1.59; 95%CI = 1.03–2.47).
2018	Johnson et al.	Population-based cohort study	1,495 college students and business professionals	Management and entrepreneurship impulsion.

*Calculated by the present authors.

CI, confidence interval; OR, odds ratio; SMD, standardized mean difference.

Sutterland et al. (2015) conducted a meta-analysis of 50 studies worldwide on the correlation between *T. gondii* and mental disorders, including 12,009 cases in the experimental group and 71,441 cases in the control group. The results showed that schizophrenia, bipolar disorder, obsessive–compulsive disorder, and addiction were significantly associated with the seropositivity of anti-toxoplasma IgG antibodies. A recent case–control study also consistent with the above observations found a higher seroprevalence among schizophrenia patients especially in suicide attempters, against the control groups, and patients with no history of suicide attempts (Akgul et al., 2021). A large-scale case–control study including 81,912 blood donors by Burgdorf et al. (2019) showed that *T. gondii* IgG seropositive rates were also significantly associated with those of schizophrenia; they also found a very weak association with traffic accident. Another meta-analytic review involving a total of 2,553 patients and 1,703 controls confirmed the association, indicating that acute psychosis was significantly associated with acute *Toxoplasma* infection (IgM seropositive), and the association was stronger for patients with chronic schizophrenia. However, first-episode psychosis was not associated with acute *Toxoplasma* infection (OR = 1.47, p = 0.181) (Monroe et al., 2015). Besides, a personality investigation of 230 women with acute *Toxoplasma* infection by Flegr et al. (2000) showed that latent toxoplasmosis may led to personality changes in the subjects, and the degree of change tended to be obvious with the extension of infection time. Two meta-analysis reports in this community showed that *T. gondii* infection increased the risk of traffic accidents, which might be related to “road rage” triggered by emotional irritation (Gohardehi et al., 2018; Sutterland et al., 2019). In addition, numerous studies demonstrated *T. gondii* as a replicated risk factor in human psychiatric disorders such as unknown temporal lobe epileptic seizure (Ngoungou et al., 2015; Uzorka and Arend, 2017; Sadeghi et al., 2019), Alzheimer’s disease (Bayani et al., 2019), cognitive impairment in several cognitive domains including short-term verbal memory and processing speed (Gale et al., 2015; de Haan et al., 2021), depression (Kamal et al., 2020), autism (Flegr and Horacek, 2019; Nayeri et al., 2020; Al et al., 2021), suicide and suicidal behavior (Kamal et al., 2020; Soleymani et al., 2020; Postolache et al., 2021), and other neurological disorders (Desmettre, 2020; Nayeri et al., 2021). Moreover, for human hosts, latent infection may also be associated with systemic changes in human personality (Lafferty, 2006). For example, Johnson et al. (2018) found that the entrepreneurial rate of *T. gondii-*positive individuals was 1.8 times higher than that of the control group (n = 197), suggesting that *T. gondii* infection may also weaken human’s awareness of danger.

These reports demonstrated a significantly elevated risk of anti-toxoplasma antibody seroprevalence among individuals diagnosed with psychiatric diseases such as schizophrenia. As a result, certain common psychiatric drugs, like haloperidol and zuclopenthixol, have been tried to clear the parasites (Fond et al., 2014). Some studies have also attempted to use antiparasitic drugs as an adjuvant intervention in the treatment of psychiatric disorders associated with *T. gondii* infection.

## Current Evidence for *T. gondii*-Induced Behavioral Changes in Animal Models

In animal models, there were a variety of research on the impact on learning, exercise, mate choice, social interaction, anxiety, exploration, and other behavior patterns. However, the conclusions of host behavioral changes are still focused on the discussion of “whether the host will increase the risk of predation,” due to the differences in experimental animal strains, *T. gondii* genotypes, and behavioral experimental methods (Worth et al., 2014). In general, chronic infection may lead to fear reduction in animal hosts, more impulsive and bold behavior, and more likelihood to be preyed upon (Tong et al., 2021). A study by Vyas et al. (2007) showed that chronically infected mice have a highly specific tendency towards cat urine odor, consistent with the previous “Fatal Attraction” hypothesis (Berdoy et al., 2000). However, the recent research by Boillat et al. (2020) indicated that the infected mice were not specific to the odor of feline predators but showed reduced fear and aversion to various predators. They also demonstrated the “parasites manipulation” of rodent hosts mainly presented as hyperactivity, impulsivity, and decreased fear and found that this “manipulation” is positively correlated with the amount of cysts loading in the host cerebral cortex.

Although the rodent model infected with *T. gondii* is one of the most studied models of parasitic behavioral manipulation (Afonso et al., 2012; Webster et al., 2013; Tan and Vyas, 2016; Meurer et al., 2020; Acquarone et al., 2021; Evangelista et al., 2021), there is still no study that statistically evaluated the tendency to be preyed upon for infected hosts, and the change pattern in daily behavior of chronically infected mice is not specifically elucidated. Besides, the behavioral effects of animal host are also correlated with the clonal lineage of *T. gondii* strains; for example, infection of inbred mice with acute virulent type I strains causes little specific behavioral disorders but severe acute sickness (Soh et al., 2013). Furthermore, key genes responsible for behavioral change in humans and mice have not yet been identified. Therefore, it is difficult to use the existing data of animal behavioral effects as a reference for analyzing the psychiatric disorder effects on humans (Webster et al., 2013).

## The Physiological Basis of These Psychiatric and Behavioral Disorders Caused by Chronic *T. gondii Infection*

### The Controversial Opinion of the Necessity for Cerebral Cysts Existence

*T. gondii* can migrate to the host brain within 7 days after invading. The mouse infection model test showed that about 10% of neurons and astrocytes and 30% of microglia could be infected by tachyzoites (Konradt et al., 2016). After tachyzoites transformed into bradyzoites, the cerebral cysts could become aggressive to persistently infect host central nervous system (CNS). At present, it is widely believed that cerebral cysts are distributed throughout the brain, despite the absence of well-targeted tropism (Berenreiterova et al., 2011; McConkey et al., 2013; Galvan-Ramirez et al., 2019; Barbosa et al., 2020). It has also been shown that cerebral cysts mainly distributed in the hippocampus (Galvan-Ramirez et al., 2019), amygdala, and cerebral cortex (Barbosa et al., 2020), while region-dependent distribution is still absent. Boillat et al. (2020) photographed the infected mouse brain tissue with fluorescence-labeled cysts supplemented by three-dimensional imaging and demonstrated that cerebral cysts were mainly distributed in the cerebral cortex, but there was still significant heterogeneity in fine localization among different infected individuals. In their study, they infected mice with three kinds of mutant strains of *T. gondii*, which could lead to a significant drop in the tissue cyst number in the infected brains, and the infected mice exhibited similar anxiety and exploration profiles as uninfected mice and displayed clear predator avoidance. Therefore, the study indicated that cerebral cyst burden correlated with the severity of behavioral changes in infected mice. While other experiments demonstrated that behavioral change of loss innate aversion to feline predator urine in the host could be also observed in mice infected with attenuated type I and low-virulence type III strains, suggesting that this behavioral change was not directly correlated with cysts load (Ingram et al., 2013). A recent study also showed that the drug Guanabenz could reverse a key behavioral change induced by latent toxoplasmosis without affecting cyst burden in the brain (Martynowicz et al., 2019). As a result, up to now, whether the specific location and existence of cerebral cysts are necessarily related with host behavioral disorders is still a controversial opinion (Martynowicz et al., 2019; Abdulai-Saiku et al., 2021).

However, brain cysts have been confirmed to cause persistent infection of nerve cells such as astrocytes, neurons, and microglia (Courret et al., 2006). In fact, brain cysts could damage neurons, change the concentration of neurotransmitters, and interfere with the process of neuroimmune regulation in the host (Ortiz-Guerrero et al., 2020). Infected mice with cerebral cysts burden showed significant behavioral disorders like diminished exploratory activity (Barbosa et al., 2020), and the neurological and behavioral abnormalities are progressive and persistent in chronically infected mice that acquired primary infection in early adulthood (Hermes et al., 2008).

### Neuroendocrine Programs and Neurotransmitter Imbalance May Act as the Physiological Basis for *T. gondii*-Induced Psychiatric and Behavioral Disorders

*T. gondii* could invade the neurons and astrocytes, accompanied by eliciting an inflammatory response in the brain (Ingram et al., 2013; Blanchard et al., 2015). The neuroinflammation response induced by pathogen infection may be a precipitating factor for behavioral changes in infected individuals. The activated inflammatory cytokines and chemokines, such as interleukin (IL)-1β, IL-6, tumor necrosis factor alpha (TNF-α), and chemokines like interferon-inducible protein 10 (IP-10) and monocytic chemotactic protein 1 (MCP-1), may recruit monocytes and macrophages into the brain, or directly act on neurons, inducing apoptosis of astrocytes and glial (Lambert et al., 2006; Ortiz-Guerrero et al., 2020), thus impairing functions of the neuron. Furthermore, altered neurotransmission levels secondary to neuroinflammation response may be responsible for the specific manipulation effects, including dopamine and other related neurotransmitters such as gamma-aminobutyric acid (GABA).

### Dopamine–Norepinephrine Hypothesis

Dysregulation of dopamine has been implicated in various human psychiatric disorders. It is also one of the most repeated findings in schizophrenia. As a result, dopaminergic signal transduction has attracted considerable attention as an important etiology to the pathology of schizophrenia (Howes and Kapur, 2009). Mcconkey et al. showed that dopaminergic cells and brain tissues encysted with cerebral cysts have increased levels of dopamine synthesis and release (McConkey et al., 2013). The processing of various physiological processes such as executive, memory, and emotional control, and psychiatric disorders in the cerebral cortex might be influenced (Prandovszky et al., 2011; Martin et al., 2015). It has also been proven that the secretion of dopamine could be enhanced *via* the releasing of cytokines such as IL-2 and IL-6 (McConkey et al., 2013).

It has been confirmed that *T. gondii* can encode two types of tyrosine hydrogenases, named as TgAAH1 and TgAAH2, respectively, which function as the rate-limiting enzymes in two dopamine synthesis pathways (Gaskell et al., 2009). Unfortunately, TgAAH2 does not affect dopamine-dependent neurobehavioral abnormalities in the host in both acute and chronic *T. gondii* infection mouse models (Afonso et al., 2017; McFarland et al., 2018). In spite of this, a recent study has shown that the degree of neuronal damage caused by *T. gondii* infection varies with the expression level of tyrosine hydrogenase (Barbosa et al., 2020). Additionally, the distribution of dopamine receptors are rather ubiquitous in the hippocampus, amygdala, cortical regions, and olfactory bulb, and in the location of *T. gondii* cysts in infected brain. All of those findings remind that continuous research in the dopamine regulatory pathway is still necessary.

Currently, correlation factors identified in host dopamine dysfunction induced by *T. gondii* infection include dopamine transporter (DAT) (McFarland et al., 2018), dopamine D1 receptor family (DRD1, DRD2, DRD4, and DRD5), MAO-A enzyme, and dopamine-mediated transduction proteins (such as DARPP-32) (Xiao et al., 2014). All of these factors lead to the imbalance of dopaminergic metabolism pathway. Moreover, the fact that anomalous accumulation of dopamine between synapses could cause various pathological processes such as increased dopamine concentration, synaptic structure disorder, proliferation of neural progenitor cells, and migration of neuron cells (Ortiz-Guerrero et al., 2020), thus may lead to the occurrence of psychiatric behavioral disorders. Alsaady et al. (2019) also found that *T. gondii* could regulate the expression of dopamine β-hydroxylase (DBH) gene, which is responsible for dopamine synthesis of NE, resulting in a decrease in NE concentration in human/rat neurons that were cultured *in vitro* and were infected with tachyzoites, but the decrease in NE in rat brain tissue was limited in males. The authors also demonstrated that this sex-specific downregulation of DBH was associated with host behavioral changes. However, Goodwin et al. (2012) found that compared to the controls, no changes in dopamine levels had been detected in mice with congenital toxoplasmosis, but they also presented behavioral changes, suggesting that other types of transmission systems may be involved in this heritable epigenetic regulation process.

### 5-HT/GABA Hypothesis

After infected with *T. gondii*, the CD^8+^/CD^4+^ T cells can secrete inflammatory cytokines such as IL-6, IL-1β, and TNF-α and stimulate microglia and astrocytes in the brain tissue (Carruthers and Suzuki, 2007). The hypothalamic–pituitary–adrenal (HPA) axis was thereby negatively regulated, resulting in the imbalance of glucocorticoids and synthesis of canine uric acid (KYNA). In particular, astrocytes could degrade tryptophan and release large amounts of KYNA to affect surrounding neurons (Notarangelo et al., 2014; McFarland et al., 2018). KYNA could inhibit the activity of glutamate receptor (NMDAR) and acetylcholine receptor (AchR), thus inhibiting the cognitive function of the infected individuals (Maddison and Giorgini, 2015). Consistent with this, the level of KYNA in schizophrenia patients is also significantly increased (Schwarcz and Hunter, 2007).

*T. gondii* can also interfere the synthesis of 5-hydroxytryptamine (5-HT) through the KYNA pathway (Mahmoud et al., 2016) and use the inhibitory neurotransmitter GABA as a carbon source for its metabolic activities (Brooks et al., 2015). *T. gondii* infection induces hypermigration of host microglia and dendritic cells through GABAergic signaling pathway, resulting in the loss of inhibitory synapses and causing dispersion of parasites in the brain parenchyma, thus may induce psychiatric symptoms such as abnormal fear response (Fuks et al., 2012; Bhandage et al., 2019; Carrillo et al., 2020). The activation of GABA receptors and L-type voltage-dependent calcium channels may participate in hijacking GABAergic signaling process (Kanatani et al., 2017).

GABA is the main inhibitory neurotransmitter in the brain; disorder of its production could generate imbalance of inhibitory and excitatory neural activities. The distribution of GABA receptors concentrated on regions that control fear and anxiety, such as the amygdala, hippocampus, and frontal lobe (Kaushik et al., 2012); therefore, it could regulate fear and anxiety behaviors by binding to receptors in the brain. Hence, GABA generation disorder may cause abnormal fear responses. For example, File et al. (1993) reported that the decreased function of GABA in the cerebral cortex and the enhanced function of 5-HT in the hippocampus may be responsible for enhanced anxiousness of rats.

Consistent with this, *T. gondii* infection could increase the distribution dispersion of a key synthetase of GABA, glutamate decarboxylase 67 (GAD67), in the brain, thus affecting the GABA synthesis process (Brooks et al., 2015). Likewise, the expression level of GAD67 in the frontal cortex was reduced in juvenile and adult mice infected with *T. gondii* (Kannan et al., 2016). Hypothesis of GABAergic function is promising for now, and the following correlation analysis of *T. gondii-*induced behavioral changes seems ready to come out. Martynowicz et al. (2019) also demonstrated that Guanabenz, an effective drug for neuroinflammation, could significantly reverse hyperactivity in C57BL/6 mice induced by latent toxoplasmosis. Therefore, behavioral disorders of the infected host are probably the result of dopamine dysfunction or dysregulation of GABA pathway. These confirmed neurophysiological changes are a better guide for further understanding the molecular mechanism from the level of gene expression regulation.

## Advances of Epigenetic Regulation in *T. gondii*-Induced Behavioral “Manipulation”

Epigenetic research technology provides opportunities for elucidating the *T. gondii* “manipulation” process. Epigenetics refers to changes reflecting on the gene’s expression level and function, but without mutants in the DNA sequence, and resulting in heritable phenotypes. It plays an important complementary role for traditional genetics and has become a hot topic in recent years. The main epigenetic regulation process includes DNA/RNA methylation, chromatin modification, and non-coding RNA. Epigenetics connects environmental factors and intrinsic genetic factor, acting as bridges and ties that make organisms more adaptable to environmental changes and external disturbance such as infection and maintain genomic stability and homeostasis. Regarding parasites including *T. gondii*, which can change the behavior of their hosts, epigenetics is more likely to reveal the mechanism of interaction between parasites and hosts.

In fact, *T. gondii* is able to affect the host transcription elements through epigenetic regulation during its invasion, affecting the expression of host genes at both the transcriptional level and the post-translational modification level, using many strategies including epigenetic enzymes, secreting epigenators into host cells, sequestering host signaling proteins, and co-opting non-coding RNAs to change gene and protein expression (Villares et al., 2020). For example, *T. gondii* can drive host epigenetic manipulation by secreting a number of effectors into the host cell in order to regulate host gene expression, especially making an effect on immune response. Braun et al. (2019) found that a member of dense-granule effectors, named as TEEGR (*Toxoplasma* E2F4-associated EZH2-inducing GeneRegulator), could associate with host transcription factors E2F3 and E2F4 when it was secreted into the host nucleus. The complex could further induce expression of a host epigenetic enzyme EZH2, then repress transcription of approximately 71 host immune system genes, through H3K27 methylation on the promoters of nuclear factor kappa B (NF-κB) target genes, thereby allowing immune evasion and parasite persisting. Although the relationship between TEEGR-EZH2 depressed genes and neuroinflammation response induced by *T. gondii* has not yet been discussed, we believe it is also an important direction in the future.

In recent years, the epigenetic research on behavioral disorders induced by *T. gondii* infection has attracted much attention and made good progress. In 2014, Hari and Vyas (2014) detected the methylation level of the arginine vasopressin gene (AVP) in the medial amygdala of the brain tissue of infected male mice and found that after infection, the promoter of host AVP was hypomethylated and the gene expression was increased, resulting in increased secretion of testosterone and eventually leading to the reduction in fear to cat urine odor in infected mice. Especially, this “fear reduction” could be reversed by systemic hypermethylation in infected animals and reproduced by directed hypomethylation of the amygdala. It has been indicated that DNA methylation is integral to the behavioral plasticity in accordance to the changing environment (Nelson et al., 2013). This research demonstrated that *T. gondii* may utilize this pre-existing phenotypic plasticity to induce host behavioral disorders by changing DNA methylation in the MePD-AVP. The research, thus, present the possibility that *T. gondii*-induced “behavioral manipulation” of the host is based on an epigenetic mechanism.

Simultaneously, testicular global methylation analysis and detection of specific methylation of crucial genes for spermatogenesis (CREM, CREB1, and HSPA1) of infected mice indicated that the overall methylation level of testicular genes in infected mice was increased, whereas hypomethylation of CpG sites in the three specific genes was detected. The findings also suggested that *T. gondii* might reduce the reproductive ability of male mice through epigenetic regulation (Dvorakova-Hortova et al., 2014). Subsequently, Singh et al. indicated that exogenous supply of testosterone in the medial amygdala could induce innate fear behavioral change in uninfected rats, similar to the effects on *T. gondii* infection. However, the methylation status of castrated rats infected with *T. gondii* did not statistically significantly change (Singh et al., 2020).

Additionally, the expression of dopamine β-hydroxylase (DBH), an enzyme that converts DA to NE, was specifically downregulated in the infected cells and male mice brain tissues. It leads to the suppression of NE and alternation of sociability and arousal (Alsaady et al., 2019). In particularly, reduction in DBH may be a specific neuron response to *T. gondii* infection, since human cytomegalovirus could not induce similar downregulation of DBH. Moreover, noradrenergic-linked behavior changes of infected animals have a high correlation between DBH expression and infection intensity. Thus, the authors indicated that how *T. gondii* downregulates DBH expression needs further clarification, and the mechanism(s) may be similar to that of vasopressin receptor by epigenetic changes (Hari and Vyas, 2014). These studies preliminary explained the molecular mechanism of chronic *T. gondii* infection-induced host dysregulation of cat urine odor in epigenetic regulation. However, this “testosterone mediated fear reduction” is restricted to male animal models. Therefore, the question of how *T. gondii* manipulates female model behavioral change through gene expression regulation strategies may focus on epigenetic regulatory pathways associated with female sex hormones such as oxytocin.

Detection of the genome-wide methylation levels and transcriptome data of retinal cell lines infected with tachyzoites also showed that two pathways were significantly interfered by *T. gondii*. The one was the dopamine-DARPP32 feedback pathway (p = 0.020), and the other was the amyloid protein pathway (p = 0.043) (Syn et al., 2018). Both of the two pathways are associated with Alzheimer’s disease, Parkinson’s disease, and schizophrenia, suggesting that there is a close correlation between host’s global methylation modification and psychiatric disorders in the process of infection. In this study, type I *RH* strain of *T. gondii* was used to establish an *in vitro* infection system in human WERI-Rb-1 eye cell line, and the two pathways could only be enriched at 6 h post-infection, whereas the reduction in amyloid precursor protein (APP) product was only detected at 18–36 h of infection. The results demonstrated that the epigenetic perturbation of host dopamine pathways and amyloid processing could be triggered by tachyzoites. Although the eye cell line is highly associated with congenital toxoplasmosis and pathological effects, research on whether bradyzoites could modulate host epigenome and induce similar dysregulation pathways in CNS or neurocytes needs to be further clarified.

Reduction level of a major mental illness-related susceptibility factor disrupted in schizophrenia (DISC1) in primary mouse cortical glial cells could enhance glial response to *T. gondii* infection (Kano et al., 2020). The epidemiological analysis results also showed that DISC1 LEU607PHE polymorphism was more common in anti-toxoplasma seropositive population. However, only one cohort in this research showed the association of anti-*T. gondii* IgG levels and the DISC1 polymorphism. Geographical locations and lifestyle may act as affecting factors, and thus, it still needs a large number of samples. As Kano’s presentation, the primary neuroglial cells isolated from C57 mice that carried LEU607PHE mutant on DISC1 were unable to interact with activating transcription factor ATF4 after infected by tachyzoites, resulting in abnormal expression of 71 downstream genes in the host. Therefore, DISC1 may be a susceptibility gene for the abnormal behavior induced by *T. gondii.* Although epigenetic manipulation has not been mentioned in this research, the regulatory process of DISC is associated with the behavioral disorders of host resistance to environmental stress (Wong and Josselyn, 2016). Since this study was confined to acute infection of glial cells *in vitro*, further interpretation of its transcriptional regulation mechanism induced by *T. gondii* and validation for its correlation with epigenetic manipulation would be a promising trial in chronic infection animal models.

Similarly, expression levels of lncRNA and microRNA (miRNA) also changed significantly after the host was infected by *T. gondii* (Bayani et al., 2019). Meanwhile, dysregulated expression of a large number of miRNAs is also an important feature of the transcriptome in patients with schizophrenia (Gale et al., 2015). Given that dopamine dysfunction is one of the most repeated findings in schizophrenia, Xiao et al. (2014) found that the transcription of miR-132, a key non-coding RNA involved in the regulation of neural development, was significantly increased in human neuroepithelioma cells infected by all three genotypes of *Toxoplasma* strains. MiR-132 was upregulated not only in brain striatum but also in the peritoneal cells of mice infected with tachyzoites of *Toxoplasma* GT1 strain (type I, virulent). The increased miR-132 was closely related to dopamine metabolic pathways (such as the downregulated expression of DRD, DRD5, MAO-A enzyme, and DARPP-32), and consistently, a 38% increase in striatal dopamine levels was detected in *Toxoplasma*-infected mice. As miR-132 could regulate both neuronal and immune functions as a “neurimmiR,” and dysfunction of dopamine signaling was also a repeated result as we described, the findings provide a promising target for revealing epigenetic mechanism(s) of behavioral disorders. However, miR-132 is a common target of a variety of pathogens, and further studies are needed to clarify whether such transcription changes exist especially in *T. gondii* infection models and whether the increase in miR-132 also appeared in the process of chronic infection in animal models by using cyst-forming type IIor type III strains.

Using small RNA sequencing, Tyebji et al. (2020) indicated that the transcription level of sperm in infected mice changed significantly, and this epigenetic regulation of sperm associating with behavioral changes could be inherited intergenerationally, but its transcriptional regulation mechanism is still unclear. Sun et al. (2020) detected the expression of lncRNAs and mRNAs in brain tissues of chronic infected BALB/c mice by using chronic infected mouse animal model and gene microarray. They found that lncRNA-11496 could increase the expression of Mef2c, a myocyte-specific enhancer factor 2c involve in synaptic formation and neuronal differentiation, by binding with HDAC2. Considering that Mef2c belongs to one of the marker genes for Alzheimer’s syndrome and also serves as a susceptibility gene for a variety of psychiatric disorders, the authors suggested that synaptic damage induced by the lncRNA11496/MEF2C/HDAC2 pathway might be a novel mechanism of toxoplasmosis-induced psychiatric disorders, while further study related to behavioral test of infected models needs to be carried out. Simultaneously, Boillat et al. (2020) showed that a general neuronal marker Rbfox3 and dopamine receptor Drd1 and Drd2 genes displayed downregulated expression in highly infected brains, suggesting a strong correlation between behavioral alterations and abnormal regulation of neuronal-related genes.

There are other studies that provided evidence of host epigenetic changes induced by *T. gondii* in other situations. For example, Yang et al. (2021) discovered that *T. gondii* infection could negatively regulate histone lysine crotonylation (Kcr) in porcine alveolar macrophages; the H2BK12 crotonylation was inhibited through epigenetic modification, and the increase in a histone decrotonylase HDAC2 expression might be responsible for this process. After infection, the HDAC2-regulated H2BK12 crotonylation could suppress NF-κB activation and promote macrophage proliferation. This finding provided a basis for host immune response regulated by epigenetic manipulation during *T. gondii* infection. Since the posttranslational modification (PTM) of histones plays a key role in epigenetic regulation and chromatin remodeling, similar PTM and gene expression changes in cyst-infected brain tissues would be another research direction to solve the mechanism of behavioral disorders induced by *T. gondii*.

Nast et al. (2020) also demonstrated that regulation of host histone marks during interferon gamma (IFN-γ) immune response could be broadly inhibited by *T. gondii*. Until now, only a few of secreting effectors have been described as able to initiate host epigenetic signal: the rhoptry protein ROP16 and ROP18 and the dense granule protein GRA15, GRA16,GRA24, TEEGR, and TgIST. ROP16 is a member of ROP2 family with protein kinase activity and secreted during early stages of invasion and phosphorylates host immune-related transcription factors. A recent research (Sabou et al., 2020) first demonstrated that ROP16 could activate an important host epigenetic regulator UHRF1, leading to epigenetic silence of *cyclin B1* gene (CCNB1) and cell cycle arrest. Simultaneously, the upregulation of DNA methyltransferases (DNMTs) and DNA hypermethylation in cells infected with *T. gondii* have also been detected. Although psychiatric behavioral disorder has not been involved in these reports, common epigenetic changes like DNA hypermethylation, PTM of histones, and abnormal expression of non-coding RNA are repeatedly discovered in different types of host cells or animal models. However, up to now, although advances of epigenetic regulation to explain the mechanism of *T. gondii-*induced mental and behavioral disorders showed an exciting achievement and reasonable orientation for discovering the mechanism, adequate epigenetic evidence associated with behavioral analysis is still lacking (see **Table 2**).

**Table 2:** Current studies concerning about *T. gondii* infection and fluctuating of host epigenetic regulation or gene expression.

Year	Author and Refs	Animal/cell model and *T. gondii* strain^*^	Detection method	Target^**^	Behavior analysis method^***^	Findings^**^
2014	Hari and Vyas	Male Wistar rats infected with Pru strain	DNA methylation	AVP promoter in the MePV	Cat odor avoidance assay	Upregulated expression of AVP leads to the increased secretion of host testosterone.
2014	Dvorakova-Hortova K et al.	Male C57Bl/6 mice infected with HIF strain	DNA methylation and quantitative analysis of CpG methylation	Testicular	None	Hypomethylation of CpG sites in CREM, CREB1 and HSPA1 genes leads to reduction of host reproductive ability.
2014	Xiao et al.	Female outbred CD-1 mice infected with GT1 strain	Profiling and analysis of miRNA	Peritoneal cells and striatum	None	Upregulated expression of miR-132 is associated with changes in dopamine receptor signaling.
2018	Syn et al.	WERI-Rb-1 cell infected with RH strain *in vitro*	DNA methylation	A retinal cell line identified genes	None	Perturbation of host dopamine-DARPP32 feedback in cAMP signaling and amyloid processing. And down-regulated expression of APP.
2019	Alsaady et al.	BALB/cAnNCrl x C57BL/6NCrlF mice infected with Pru strain	RNA sequencing	Transcriptome analysis of PC12 cells	OF and Sociability	Downregulation of DBH specifically in the infected male mice leads to the NE suppression and alternation of sociability and arousal.
2020	Singh et al.	Male Wistar rats infected with Pru strain	DNA methylation	AVP promoter	Cat odor avoidance assay and OF	Upregulation of testosterone within MeA leads to the reduction in innate fear.
2020	Kano et al.	Human lymphoblastoid cells and mouse glial cells infected with RH-2F strain *in vitro*	RNA sequencing	Gene expression assay in cells with DISC1 607 Phe/Phe variant or in native cells	None	Abnormal expression of DISC1 altered 71 downstream genes upon *T. gondii* infection
2020	Tyebji et al.	Inbred C57BL/6J mice infected with Pru strain	RNA sequencing	Sperm small RNA level of F1 and F2 generation of *T. gondii*-infected male mice	OF, LDB, EZM, SP, Y-maze, 3-CSSR, MT, NOR, FST	Small RNA profiles of the F0 sperm were altered by *T. gondii* infection and associated with behavioral changes in the F2 offspring.
2020	Sun et al.	Female BALB/c mice infected with TgCtwh6 strain	LncRNAs-mRNAs microarray assay	LncRNAs-mRNAs expression in the brains of infected mice	None	The expression of lncRNA-11496 was downregulated in the chronically infected mice.
2020	Boillat et al.	Male B6CBAF1/J mice infected with ME49 strain	RNA Sequencing	Transcriptome of infected mice brain	OF, HI, EPM, EXP, HB, NOR, BA, Sociability, and PA.	Upregulated genes: IL-27, Alox5ap, IFN-g, IL-12b, TNF-α, Gfap, Ido1, and Nos2. Downregulated genes: a general neuronal marker Rbfox3, dopamine receptor Drd1 and Drd2.
2021	Yang et al.	Porcine alveolar macrophages infected with RHΔ hxgprt strain *in vitro*	ChIP and ChIP-Seq Assays	Histone of porcine alveolar macrophages	None	Inhibition of H2BK12 crotonylation and suppression of epigenetic regulation and NF-kB activation, and promotion of macrophage proliferation.
2020	Sabou et al.	Human trophoblast cell line BeWo and astrocytic cell line U-118MG infected with RH strain	ChIP analysis and methylation analysis	Histone and DNMTs	None	Upregulation of DNMTs and DNA hypermethylation. Deacetylation and methylation of histone H3 surrounding the CCNB1promoter to epigenetically silence its transcriptional activity.

*The genotype of T. gondii strain: Pru-Prugniuand strain (type II, moderated virulent cyst-forming); GT1 strain (type I, virulent); RH and RH-2F strain (type I, virulent); CTG strain (type III, avirulent cyst-forming); HIF strain (type II, avirulent cyst-forming); TgCtwh6 strain (Chinese 1 hybrid subtype, moderated virulent cyst-forming); ME49 strain (type II, moderated virulent cyst-forming).

**AVP, arginine vasopressin gene; MePV, medial amygdala; APP, amyloid precursor protein; DBH, dopamine β-hydroxylase; NE, norepinephrine; DISC1, disrupted in schizophrenia; Nos2, nitric oxide synthase; ChIP, chromatin immunoprecipitation; DNMTs, DNA methyltransferases; CCNB1, cyclin B1 gene.

***OF, open field test; PPI, prepulse inhibition of the startle refex; HI, hand investigation; EPM, elevated plus-maze; EXP, general exploration; HB, holeboard; NOR, novel object recognition; BA, bobcat aversion; PA, predator aversion; LDB, light–dark box test; EZM, elevated-zero maze; SP, sucrose preference test; 3-CSSR, three-chambered sociability and social recognition; MT, memory test; NOR, novel object recognition test; FST, forced swim test.

## Summary and Prospect

Therefore, until now, there are opportunities and challenges both in the research of host mental and behavioral disorders induced by chronic *Toxoplasma* infection. First, for humans, more than 200 serologic-testing-related reports have indicated the correlation between latent toxoplasmosis and a given psychiatric disorder such as schizophrenia, even taking negative factors like publication bias, sampling bias related to the geographical region, validity of seropositivity, and study design limitations (Johnson and Koshy, 2020). However, we still lack a direct evidence, such as anatomical evidence, to evaluate the causal relationship between chronic infection and psychiatric disorders. Second, for rodent models, severity of behavioral change induced by *T. gondii* was also different with the differences in parasite and mouse strains. For instance, C57Bl/6 mice may be a *T. gondii*-infected encephalitis- susceptible strain while BALB/c mice may be a encephalitis-resistant strain (David et al., 2016). In spite of this, the reduction in natural fear induced by *T. gondii* infection in mice was still the most repeatable experimental result based on previous studies, eliminating the influencing factors of animal strain type. Third, although the effects of neuroinflammation caused by *T. gondii* infection have been repeatedly emphasized and evaluated, they are not interested in this review. For one reason, the role of cysts participating in the “*T. gondii* manipulation” has not been determined; for another, the behavioral disorders induced by neuroinflammation are typically generalized, whereas disorders induced by parasites were likely have a particular tendency. We still lack direct causal evidence as well. Thus, epigenetics may serve as the most reliable executive for explaining how an exogenous stimulus factor constantly impact on host phenotypes and its offspring. However, there is still no identified responsible factors for this manipulation process in a large sample of humans with psychiatric disorders. Fortunately, the advances of DA-NE regulation pathways, testosterone-mediated mechanism, or related lncRNAs and microRNAs may provide an opportunity to discover ideal targets for drug treatment.

The research results on chronic *Toxoplasma* infection and host psychiatric behavioral disorders are not only helpful to understand the pathogenesis of schizophrenia and other important psychiatric diseases from the perspective of pathogen infection and immunity, but it can also help to find new drug targets and further explore the molecular mechanism of host behavioral changes caused by *T. gondii*. It is also useful for developing new measures to effectively prevent and control the complication of the two combined diseases. It even provides relevant theoretical basis for ecology (life history change, ecological community evolution), sociology (group personality change, social mode change), psychology (introversion/extroversion, impulse/addiction), and other applied disciplines.

## Author Contributions

KY and CX conceived and wrote the majority of the manuscript, GZ and HX contributed mechanism and epigenetic related aspects to the manuscript. All authors contributed to the article and approved the submitted version.

## Funding

The manuscript was supported by Taishan Scholars Project (tsqn202103186), Medicine and Health Science Technology Development Plan of Shandong Province (No. 202101050270), Academic Promotion Programme of Shandong First Medical University (No.2019QL005), and the Innovation Project of Shandong Academy of Medical Sciences.

## Conflict of Interest

The authors declare that the research was conducted in the absence of any commercial or financial relationships that could be construed as a potential conflict of interest.

## Publisher’s Note

All claims expressed in this article are solely those of the authors and do not necessarily represent those of their affiliated organizations, or those of the publisher, the editors and the reviewers. Any product that may be evaluated in this article, or claim that may be made by its manufacturer, is not guaranteed or endorsed by the publisher.
